# Anti-Müllerian hormone levels and risk of type 2 diabetes in women

**DOI:** 10.1007/s00125-020-05302-5

**Published:** 2020-10-13

**Authors:** Renée M. G. Verdiesen, N. Charlotte Onland-Moret, Carla H. van Gils, Rebecca K. Stellato, Annemieke M. W. Spijkerman, H. Susan J. Picavet, Frank J. M. Broekmans, W. M. Monique Verschuren, Yvonne T. van der Schouw

**Affiliations:** 1 Julius Center for Health Sciences and Primary Care, University Medical Center Utrecht, Utrecht University, Utrecht, the Netherlands; 2 Centre for Nutrition, Prevention and Health Services, National Institute for Public Health and the Environment (RIVM), Bilthoven, the Netherlands; 3 Department of Reproductive Medicine and Gynecology, University Medical Center Utrecht, Utrecht University, Utrecht, the Netherlands

**Keywords:** AMH, Anti-Müllerian hormone, Longitudinal, Reproductive ageing, Trajectories, Type 2 diabetes, Women

## Abstract

**Aims/hypothesis:**

Given its role in ovarian follicle development, circulating anti-Müllerian hormone (AMH) is considered to be a marker of reproductive ageing. Although accelerated reproductive ageing has been associated with a higher risk of type 2 diabetes, research on the relationship between AMH and type 2 diabetes risk is scarce. Therefore, we aimed to investigate whether age-specific AMH levels and age-related AMH trajectories are associated with type 2 diabetes risk in women.

**Methods:**

We measured AMH in repeated plasma samples from 3293 female participants (12,460 samples in total), aged 20–59 years at recruitment, from the Doetinchem Cohort Study, a longitudinal study with follow-up visits every 5 years. We calculated age-specific AMH tertiles at baseline to account for the strong AMH–age correlation. Cox proportional hazards models adjusted for confounders were used to assess the association between baseline age-specific AMH tertiles and incident type 2 diabetes. We applied linear mixed models to compare age-related AMH trajectories for women who developed type 2 diabetes with trajectories for women who did not develop diabetes.

**Results:**

During a median follow-up of 20 years, 163 women developed type 2 diabetes. Lower baseline age-specific AMH levels were associated with a higher type 2 diabetes risk (HR_T2vsT3_ 1.24 [95% CI 0.81, 1.92]; HR_T1vsT3_ 1.62 [95% CI 1.06, 2.48]; *p*_trend_ = 0.02). These findings seem to be supported by predicted AMH trajectories, which suggested that plasma AMH levels were lower at younger ages in women who developed type 2 diabetes compared with women who did not. The trajectories also suggested that AMH levels declined at a slower rate in women who developed type 2 diabetes, although differences in trajectories were not statistically significant.

**Conclusions/interpretation:**

We observed that lower age-specific AMH levels were associated with a higher risk of type 2 diabetes in women. Longitudinal analyses did not show clear evidence of differing AMH trajectories between women who developed type 2 diabetes compared with women who did not, possibly because these analyses were underpowered. Further research is needed to investigate whether AMH is part of the biological mechanism explaining the association between reproductive ageing and type 2 diabetes.

**Figure:**
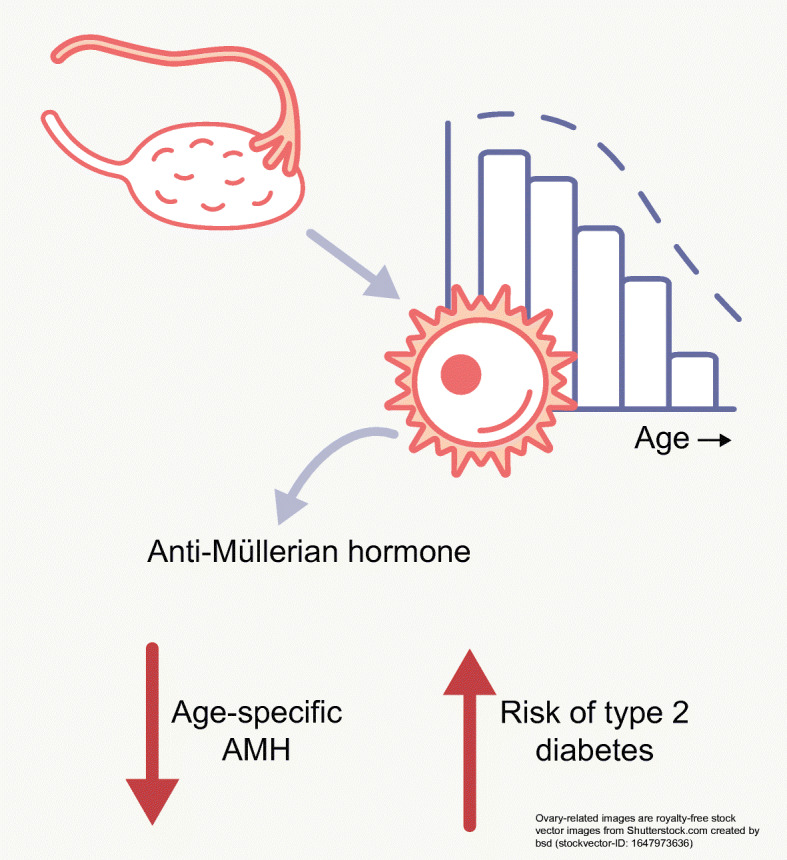
Graphical abstract

**Electronic supplementary material:**

The online version of this article (10.1007/s00125-020-05302-5) contains peer-reviewed but unedited supplementary material, which is available to authorised users.

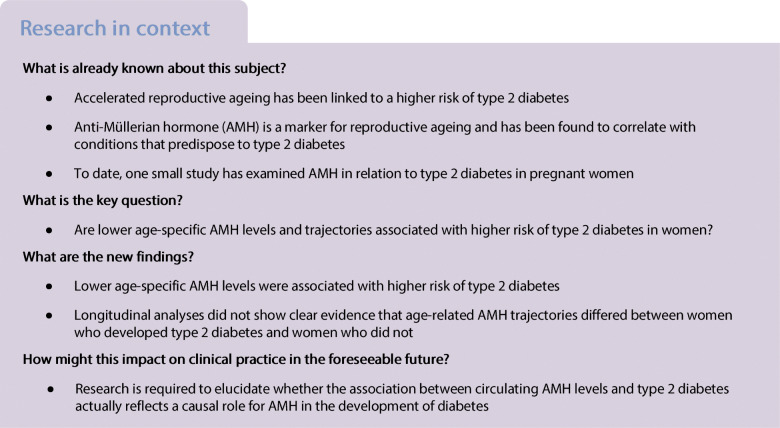



## Introduction

Female reproductive ageing has been associated with risk of chronic diseases, including type 2 diabetes, in later life [1]. Women with an earlier menopause have been found to be at a higher risk of postmenopausal type 2 diabetes [2]. This association appears to be independent from the effect of BMI [3, 4]. Yet, the biological mechanisms underlying the association between reproductive ageing and type 2 diabetes remain to be established. A potential causal candidate explaining this association is anti-Müllerian hormone (AMH), a gonadal hormone expressed by early-stage ovarian follicles in premenopausal women [5]. From birth onwards, the ovarian follicle pool decreases until menopause [6]. Accordingly, circulating AMH levels decline with age until they become undetectable after menopause. AMH can therefore be used as a marker for reproductive ageing in women [7, 8].

To date, the relationship between circulating AMH and type 2 diabetes has been examined in one small study in pregnant women [9]. Several studies investigated AMH in relation to conditions, such as insulin resistance, that predispose to type 2 diabetes but their results are inconsistent [10–14]. Furthermore, most of these studies had a cross-sectional design and/or included only women with polycystic ovary syndrome (PCOS). As a result, reverse causation could not be excluded in previous studies and generalisability of their results to healthy women is limited.

Therefore, the aim of the current study was to investigate the association between AMH and type 2 diabetes using data from women in the population-based Doetinchem Cohort Study. Specifically, we investigated associations between age-specific AMH levels at baseline of the cohort and age-related AMH trajectories and incident type 2 diabetes.

## Methods

### Study population

The Doetinchem Cohort Study is an ongoing prospective cohort study, which has been described in more detail previously [15, 16]. Briefly, the Doetinchem Cohort Study included 3641 men and 4128 women, aged 20–59 years at recruitment, who were randomly selected from the municipal register of Doetinchem, the Netherlands, between 1987 and 1991. Every 5 years, study participants are invited for a follow-up visit during which physical examinations are conducted, extensive questionnaires are completed and blood samples are collected. Invitations for the follow-up visits are sent irrespective of attendance at previous follow-up rounds. The Doetinchem Cohort Study received approval from the Medical Ethics Committee of the Netherlands Institution of Applied Scientific Research and all study participants signed an informed consent prior to study inclusion. For the current study we only used data from female participants (median age at recruitment 39 years, range 20–59 years) from examination Round 1 (baseline 1987–1991) to examination Round 5 (2008–2012).

### Exclusion criteria

**Fig. 1:**
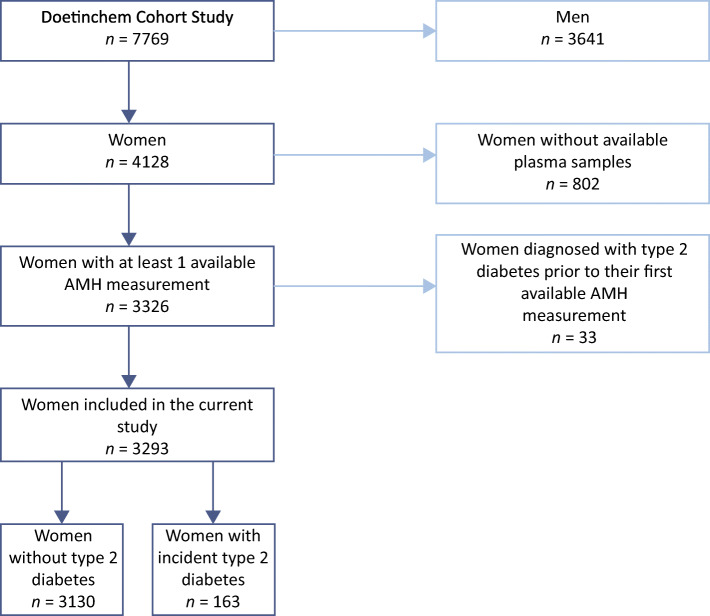
Flow chart for study population

For 3326 of the 4128 female participants in the Doetinchem Cohort Study, at least one AMH measurement was available for any of the five included examination rounds. For this study we excluded women who were diagnosed with diabetes prior to their first available AMH measurement (*n* = 33) (Fig. 1). We included data for the remaining 3293 women in subsequent analyses. The number of women with an AMH measurement per examination round was 3104, 2888, 2488, 2305 and 2038 for Rounds 1, 2, 3, 4 and 5, respectively.

### AMH measurements

Approval for AMH measurements was given by the Ethical Committee for Biobank Studies of the University Medical Center Utrecht. Details of these measurements and sample storage conditions have been described previously [17, 18]. In short, AMH was measured in all available plasma samples, collected from baseline to examination Round 5, from each female study participant. Missing AMH measurements were the consequence of either non-attendance at certain follow-up visits, no consent to blood draw at the particular examination, depletion of plasma samples because of other blood measurements, or an occasional unsuccessful AMH measurement. AMH was measured using the picoAMH ELISA (Ansh Labs, Webster, TX, USA) in the Ansh Labs laboratory. This AMH assay has a lower detection limit of 0.013 pmol/l. AMH measurements below the limit of detection were set to half this value (0.007 pmol/l).

### Covariates

Data on age at blood collection (years), educational attainment (low, middle, high), current smoking (yes, no), alcohol consumption (glasses/day), physical activity (inactive, active), parity (nulliparous, parous), current oral contraceptive use (yes, no), ever hormone replacement therapy (HRT) use (yes, no) and menopausal status (premenopausal, postmenopausal) were collected through questionnaires. Time-varying data was available for age at blood collection, BMI, current smoking, alcohol consumption, physical activity, hypertension, total cholesterol, current oral contraceptive use and menopausal status.

Educational attainment was classified using the following categories: primary education up to completing intermediate vocational education (low); up to higher secondary education (middle); and higher vocational education and university (high) [19]. Women were classified as current smokers if they reported smoking on average ≥1 cigarette per month. Total alcohol consumption (glasses/day) was calculated in women who reported consuming on average more than one glass of alcohol per week. Physical activity was assessed using the validated Cambridge Physical Activity Index [20]. Because data on physical activity at baseline was completely missing, we assumed that physical activity at baseline was equal to data from Round 2. Questions on current and ever HRT use were only included in questionnaires from Rounds 2–5. Consequently, women were classified as ever HRT users when they reported HRT use on at least one of these questionnaires. Women who reported no HRT use on any of the questionnaires were classified as never HRT users. Menopausal status was assessed as previously described [17]; women who had amenorrhea for at least 12 consecutive months were considered postmenopausal. Women who underwent a bilateral oophorectomy were considered postmenopausal from the moment they had surgery. Menopausal status was set to ‘missing’ for women who had a hysterectomy without bilateral oophorectomy and for current oral contraceptive users, and imputed subsequently as described in the statistical analyses section. We imputed menopausal status in current oral contraceptive users because Dutch guidelines state that oral contraceptive use is preferable in perimenopausal women with vasomotor complaints. In addition, women with birth control wishes use oral contraceptives as the preferred method up to the age of 52.

BMI (kg/m^2^) was calculated using standardised weight and height measurements obtained during physical examinations. Hypertension (yes, no) was classified according to the guidelines of the WHO (systolic BP ≥140 mmHg and/or diastolic BP ≥90 mmHg) and/or use of BP-lowering medication. Total cholesterol (mmol/l) was measured in non-fasting EDTA–plasma until 1998 and in serum from 1998 onwards, using standardised enzymatic methods [16].

### Ascertainment of type 2 diabetes

Women reporting that they had been diagnosed with diabetes for the first time at Rounds 2–5 were classified as incident type 2 diabetes cases. In addition, non-fasting glucose measurements were available in Rounds 2–5, and women with at least one glucose measurement ≥11.1 mmol/l were also classified as incident cases. Previous research has shown that 86% of the self-reported diabetes cases in the Doetinchem Cohort Study could be confirmed by general practitioner or pharmacy registries [21]. In total, we identified 163 incident type 2 diabetes cases over a median follow-up period of 20 years. For women who reported their age at diabetes diagnosis, we set their diagnosis date to the first day of January of the corresponding year. For the remaining women, we set their diagnosis date to the first day of January of the year in which the examination during which they first reported to have been diagnosed with diabetes or at which their glucose was ≥11.1 mmol/l took place.

### Statistical analyses

We calculated age-specific baseline AMH tertiles using general linear modelling with the Cole and Green, Lambda, Mu and Sigma (CG–LMS) method [22] (R package ‘gamlss’, version 5.1-2 [23]), as previously described [24]. _log_AMH (natural logarithm) at examination Round 1 was modelled over age using splines because of the non-linear decline in AMH with increasing age. Previous analyses showed that this model fits the AMH data in the Doetinchem Cohort Study well [17]. The CG–LMS method allows for estimation of the distribution of AMH at every age, and corresponding percentile values (for 33.3% and 66.7%) were used to create age-specific tertiles. Accordingly, women could be classified as having either low (first age-specific tertile), normal (second age-specific tertile) or high (third age-specific tertile) AMH levels given their age.

Characteristics for women with an available AMH measurement at baseline (*n* = 3104) were described using medians (IQR) or percentages (*n*). We summarised these baseline characteristics by age-specific AMH tertiles. In addition, we compared baseline characteristics and the proportion of incident diabetes cases between women with and without an AMH measurement at each round, to assess whether missing AMH measurements were potentially associated with these characteristics.

Missing values for baseline age-specific AMH tertiles and baseline and time-varying covariates were imputed with multiple imputation (100 iterations, ten imputed datasets) using the R package ‘mice’ (version 3.3.0) [25] (ESM Methods). We based the number of imputed datasets on the average proportion of missing values on variables included in the association analyses (8.0%), as recommended previously [26]. Subsequent regression analyses were performed in each imputed dataset; regression coefficients and 95% CIs were pooled according to Rubin’s Rule of combination [27] using the pool function in ‘mice’.

#### Baseline age-specific AMH tertiles and type 2 diabetes risk

We assessed associations between baseline age-specific AMH tertiles and incident type 2 diabetes by estimating HRs and 95% CIs from Cox proportional hazards models. We used follow-up time in years as underlying time scale (t_0_ represented baseline examination; t_max_ represented either date on which participant last attended an examination or date at diabetes diagnosis), and adjusted models for known risk factors for type 2 diabetes and reproductive factors. Fully adjusted models included the following baseline variables: age; BMI; educational attainment; current smoking; alcohol consumption; physical activity; hypertension; total cholesterol; current oral contraceptive use; parity; and menopausal status. We visually checked the proportional hazards assumption using scaled Schoenfeld residuals and statistically tested it using the cox.zph function in R (R package ‘survival’, version 2.44-1.1 [28]), which consistently indicated that the proportional hazards assumption was not violated.

#### Mean AMH trajectories in women who develop type 2 diabetes compared with women who do not

To assess whether age-related AMH trajectories differed between women with and without incident type 2 diabetes, we used linear mixed models (R package ‘nlme’, version 3.1-139 [29]). AMH trajectories were constructed using available measurements from examination Rounds 1–5. We included non-imputed AMH values in the linear mixed model analyses, as these analyses provide unbiased estimates when outcomes are missing at random [30]. Imputed values were included for the covariates described below. We excluded AMH measurements after diabetes diagnosis. In women with incident type 2 diabetes, the earliest age at which AMH was measured was 21.4 years. Accordingly, we excluded 79 AMH measurements that were available at earlier ages for women without diabetes, as differences in AMH trajectories between both groups cannot be assessed at ages for which no measurements were available in one of the groups. Two women without diabetes were completely excluded from these analyses due to these excluded measurements. In addition, one woman with incident type 2 diabetes was excluded from our longitudinal analyses because no AMH measurements were available before her diagnosis. As a result, we included data from 3290 women, among which there were 162 incident cases of type 2 diabetes, in our longitudinal analyses. In total, we included 12,460 AMH measurements performed in the period from baseline until diabetes diagnosis or last-attended examination round. Of these measurements, 4587 (36.8%) were below the limit of detection (<0.013 pmol/l).

Models included repeated _log_AMH levels as dependent variable and age in years, modelled with natural splines (2 knots, 36 and 45 years; upper boundary, 65 years), as the underlying timescale. To assess whether models including incident type 2 diabetes status (yes, no) and interaction terms of this case variable and the spline terms were a better fit to the data compared with models without these variables, a global likelihood ratio test was applied [31] using the testModels function (method ‘D3’) implemented in R package ‘mitml’ (version 0.3-7 [32]). Linear mixed models additionally included the following fixed effects: age at blood collection; BMI; educational attainment; current smoking; alcohol consumption; physical activity; hypertension; total cholesterol; current oral contraceptive use; parity; and menopausal status. Except for educational attainment and parity, all included covariates were time-varying. We also included random intercepts and random slopes for age for each woman. We used the estimated fixed effects from these models to calculate predicted geometric mean AMH trajectories adjusted for the described potential confounders over age per imputation set. Predicted AMH trajectories and corresponding 95% CIs were pooled using Rubin’s Rule. All analyses were performed in R, version 3.6.0 [33].

#### Sensitivity analyses

To rule out a potential effect of undiagnosed type 2 diabetes on AMH measurements included in our analyses, we repeated our main analyses after excluding AMH measurements in samples collected within 2 years prior to diabetes diagnosis (*n* = 8). We also explored how imputation of baseline age-specific AMH tertiles influenced our survival analyses through excluding women with missing AMH data at baseline (*n* = 189)*.* Current HRT use has been shown to affect both AMH levels and risk of diabetes. As current HRT use was not assessed at Round 1, we could not model this variable as a time-varying covariate. Instead, we assessed a potential effect of HRT use on our main results by performing analyses excluding women who reported any use of HRT at Rounds 2–5 (*n* = 1490 on average over ten imputed datasets). In addition, we performed sensitivity analyses in which we excluded women who never reported having had regular menstrual cycles during follow-up (*n* = 268), as this could be an indication that these women had PCOS. Although an irregular menstrual cycle in itself is not sufficient to diagnose PCOS, no other data was available that allowed us to assess whether women potentially had PCOS.

## Results

Characteristics of the women with an available AMH measurement at baseline are presented by age-specific tertile in Table 1. Women in the middle and highest tertiles were younger, more often premenopausal, less likely to ever have used HRT, and more physically active than women in the lowest age-specific AMH tertile. In addition, women in the highest age-specific tertile were more likely to be highly educated and consume more alcohol but were less likely to be a current oral contraceptive user, current smoker or to be hypertensive compared with women in the middle and lowest age-specific AMH tertiles. Baseline characteristics and the proportion of incident diabetes cases were mostly comparable between women with and women without a missing AMH measurement, for each of the five examination rounds (ESM Table 1).

**Table 1:** Characteristics of women with an available AMH measurement at baseline of the Doetinchem Cohort Study (*n* = 3104) presented by age-specific AMH tertiles

Characteristic	Lowest age-specific AMH tertile (*n* = 907)	Middle age-specific AMH tertile (*n* = 1184)	Highest age-specific AMH tertile (*n* = 1013)
AMH, pmol/l	0.21 (0.01–5.38)	8.90 (0.85–19.29)	26.85 (7.07–45.31)
Age, years	42.0 (32.6–51.4)	38.5 (31.6–46.2)	39.1 (32.1–45.8)
BMI, kg/m^2^	23.7 (21.7–26.3)	23.7 (21.7–26.2)	23.3 (21.4–25.7)
Educational attainment^a^			
Low	70.9 (641)	69.3 (818)	64.0 (646)
Middle	16.7 (151)	19.1 (226)	21.0 (212)
High	12.4 (112)	11.6 (137)	15.0 (152)
Reproductive factors			
Parous, yes	77.1 (699)	75.1 (889)	78.6 (796)
Premenopausal^a^	73.9 (588)	83.0 (906)	96.2 (917)
Current OC use^a^	29.8 (269)	27.6 (327)	18.8 (190)
Ever HRT use^a,b^	36.5 (206)	27.2 (190)	27.8 (184)
Lifestyle factors			
Current smoker, yes^a^	35.2 (319)	35.4 (419)	30.3 (307)
Current alcohol consumption^a^			
No	20.7 (188)	19.8 (235)	17.6 (178)
<1 glass/week	31.7 (287)	31.4 (372)	29.8 (302)
≥1 glass/week	47.6 (431)	48.7 (576)	52.6 (532)
Physical activity^a,c^			
Inactive	31.5 (224)	26.2 (242)	27.0 (225)
Active	68.5 (486)	73.8 (682)	73.0 (608)
Total cholesterol, mmol/l^a^	5.4 (4.7–6.2)	5.3 (4.6–6.0)	5.2 (4.6–5.8)
Hypertension, yes	15.4 (140)	14.4 (171)	10.8 (109)

Data are presented as median (IQR) or percentage (*n*)

^a^Missing values (*n*): educational attainment (9); menopausal status (263), current oral contraceptive use (7), ever HRT use (1179), current smoking (1), current alcohol consumption (3), physical activity (637), total cholesterol (1)

^b^Ever variable presented because of absent data on HRT use at baseline

^c^Physical activity at examination Round 2 due to absent data on physical activity at baseline

AMH, anti-Müllerian hormone; OC, oral contraceptive; HRT, hormone replacement therapy

### Baseline age-specific tertiles and risk of type 2 diabetes

We observed that women with lower age-specific AMH levels had a higher risk of type 2 diabetes (HR_T2vsT3_ 1.24 [95% CI 0.81, 1.92]; HR_T1vsT3_ 1.62 [95% CI 1.06, 2.48]; *p*_trend_ across tertiles = 0.02) (Table 2). Sensitivity analyses excluding AMH measurements performed in plasma samples collected within 2 years prior to diabetes diagnosis and analyses excluding women with missing AMH data at baseline did not change these results (Table 2). Exclusion of women with potential PCOS did not considerably change effect estimates either, although associations were no longer statistically significant (Table 2). Exclusion of women who ever had HRT resulted in wider CIs and decreased effect estimates for both the first and second age-specific AMH tertile.

**Table 2:** HRs (95% CIs) for the association between baseline age-specific AMH tertiles and risk of type 2 diabetes in women of the Doetinchem Cohort Study

Population	Lowest age-specific AMH tertile	Middle age-specific AMH tertile	Highest age-specific AMH tertile (reference)	*p* value for trend
Total study population (*n* = 3293, 163 cases)	1.62 (1.06, 2.48)*	1.24 (0.81, 1.92)	1.00	0.02
Exclusion of AMH measurements within 2 years prior to type 2 diabetes diagnosis (*n* = 3285, 155 cases)	1.55 (1.00, 2.40)*	1.19 (0.76, 1.85)	1.00	
Exclusion of women with a missing AMH measurement at baseline (*n* = 3104, 148 cases)	1.62 (1.04, 2.52)*	1.29 (0.83, 2.00)	1.00	
Exclusion of women who ever used HRT (*n* = 1803, 95 cases)^a^	1.26 (0.72, 2.20)	0.74 (0.41, 1.32)	1.00	
Exclusion of women who potentially had PCOS (*n* = 3025, 138 cases)	1.57 (0.97, 2.54)	1.13 (0.71, 1.83)	1.00	

Cox proportional hazards models were adjusted for the following baseline variables: age (years), parity (nulliparous, parous), current oral contraceptive use (yes, no), menopausal status (premenopausal, postmenopausal), BMI (kg/m^2^), educational attainment (low, middle, high), current smoking (yes, no), alcohol consumption (glasses/day), physical activity (inactive, active), hypertension (yes, no), total cholesterol (mmol/l)

^a^Numbers differed between imputation sets, as the variable ever HRT use itself was imputed; presented numbers are average sample sizes and average numbers of cases

*p <0.05

### Mean AMH trajectories in women who are diagnosed with type 2 diabetes compared with women who are not

**Fig. 2:**
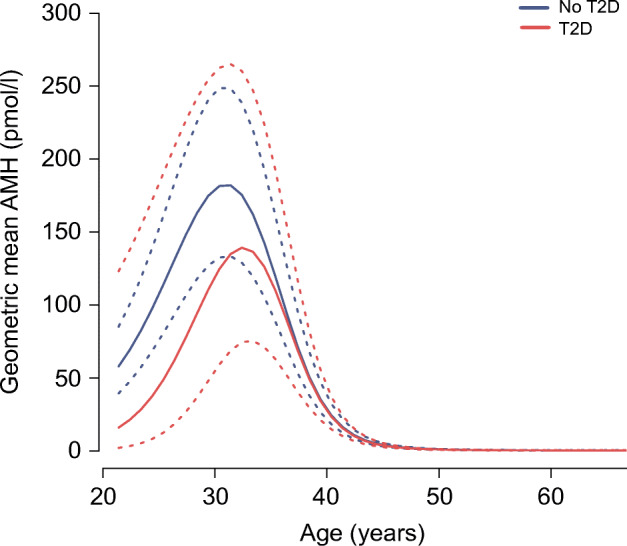
Predicted geometric mean AMH trajectories (pmol/l) (solid lines) and 95% CIs (dashed lines) over age in women who were diagnosed with type 2 diabetes compared with women who were not diagnosed with type 2 diabetes during follow-up. Plots show average predicted AMH trajectories across ten imputed datasets. Trajectories are adjusted for current oral contraceptive use, current smoking, BMI, menopausal status, alcohol consumption, physical activity, hypertension, total cholesterol, parity and educational level. T2D, type 2 diabetes

On average, 3.8 AMH measurements were available per woman. Figure 2 presents predicted geometric mean AMH trajectories in incident type 2 diabetes cases and women without type 2 diabetes averaged across the ten imputation sets. This plot suggests that AMH levels were lower until approximately 37 years of age and that from the age of 30 years onwards AMH levels declined more slowly in women who developed type 2 diabetes compared with women who did not develop type 2 diabetes. However, neither the type 2 diabetes case variable nor interaction terms of this case variable with splines for age were statistically significant (ESM Table 2). Comparing models including these diabetes variables with models that did not include them did not indicate that age-related AMH trajectories differed between women with and without type 2 diabetes either (*p* value global likelihood ratio test = 0.58). Exclusion of AMH measurements within 2 years prior to diagnosis, exclusion of women who reported ever having used HRT and exclusion of women who potentially had PCOS did not change these results (ESM Table 2).

## Discussion

In this prospective cohort study we observed that lower age-specific AMH levels were associated with a higher risk of type 2 diabetes in women. Longitudinal analyses that included multiple AMH measurements per woman did not show clear evidence of a difference in age-related trajectories between women with and without incident diabetes, possibly because of the limited number of AMH measurements at younger ages, particularly in women diagnosed with type 2 diabetes.

The main strength of this study is that we investigated the association between age-specific AMH and age-related AMH trajectories and risk of type 2 diabetes in women in a large longitudinal population-based cohort study with a median follow-up of 20 years. To date, just one small study (*n* = 69) examined AMH in relation to type 2 diabetes in women, and only included pregnant women [9]. Additional strengths of the current study are its time-varying information on AMH as well as a wide array of potential confounders, including BMI. Nevertheless, residual confounding cannot be ruled out completely.

A potential limitation of this study is that type 2 diabetes case ascertainment was based on self-report and non-fasting glucose measurements. Accordingly, we made an assumption about the date of type 2 diabetes diagnosis, which we set to the first day of January of the year in which a woman first reported that she had been diagnosed with diabetes and/or in which her glucose was ≥11.1 mmol/l. This approach may have resulted in some misclassification, although diagnosis dates obtained from hospital discharge or general practitioner registries are not precise either, because diabetes develops over several years. However, sensitivity analyses in which we excluded AMH measurements performed in plasma samples collected within 2 years prior to the assumed type 2 diabetes diagnosis date did not change our findings, suggesting that our assumption did not induce reverse causation bias. Furthermore, previous research has shown that most of the self-reported diabetes cases in the Doetinchem Cohort Study (86%) could be verified with data from general practitioner or pharmacy registries [21].

The only previous study examining AMH in relation to type 2 diabetes in women [9] observed no difference in AMH levels between women with type 2 diabetes, women with gestational diabetes and a healthy control group of women during the second and third trimester of pregnancy. The generalisability of this finding may be limited, as it has been suggested that circulating AMH levels temporarily drop during late-stage pregnancy [34]. In line with our results, a previous study in men observed a lower risk of type 2 diabetes in overweight individuals with higher AMH levels [35]. In men, AMH is produced by Sertoli cells and also decreases with increasing age [36], although to a lesser degree than in women. Lower AMH levels have also been observed in men with the metabolic syndrome [37] and in obese boys with insulin resistance [38], conditions that are both associated with an increased risk of type 2 diabetes. In women, lower AMH levels have also been reported to correlate with higher HOMA-IR [11] and higher fasting insulin [39], although other studies could not replicate this [10, 13, 40] (see de Kat et al [41] for a more detailed discussion). Similarly, results of studies on the relationship between AMH and conditions that predispose to type 2 diabetes in women diagnosed with PCOS are inconsistent [12, 14, 41].

Because AMH levels are higher in women with PCOS [42], and these women are at an increased risk of type 2 diabetes [43], we performed a sensitivity analysis in which we excluded women who potentially had PCOS. Based on the positive associations between AMH and PCOS and between PCOS and type 2 diabetes, we hypothesised that if PCOS was a confounder in our analyses, we would observe an even lower risk of type 2 diabetes in women with higher AMH levels after exclusion of those with PCOS. However, our effect estimates did not change. A likely explanation for this is that we classified women as potentially having PCOS when they reported never having had regular menstrual cycles, whereas in practice PCOS is diagnosed based on a set of criteria that additionally include clinical and/or biochemical hyperandrogenism and/or polycystic ovaries [44]. Future studies including data on actual PCOS diagnosis should indicate whether PCOS acts as confounder in the observed association between AMH and type 2 diabetes.

Given its role in ovarian follicle development and its expression in these follicles [5], AMH is considered to be a proxy for ovarian ageing and, accordingly, lower AMH levels have been associated with an earlier age at menopause [45]. Previous studies observed that an earlier age at menopause was associated with a higher risk of type 2 diabetes [2–4], which is in accordance with our results. However, the question remains as to whether ovarian ageing is indeed causally associated with risk of diabetes or whether residual confounding by biological ageing influenced our and previous findings. Future studies including data on both proxies for ovarian (e.g. AMH and/or age at menopause) and biological ageing (e.g. epigenetic clock) may provide more insight into this matter. In addition, functional studies may investigate if AMH signalling actually takes place in the pancreas and how this might be related to the pathophysiology of type 2 diabetes, since the receptor through which AMH signals (AMHR2) is expressed in pancreatic tissue [46].

In conclusion, we observed that women with lower age-specific AMH levels were at a higher risk of type 2 diabetes. Longitudinal analyses also indicated that AMH levels may be lower in women who develop type 2 diabetes compared with women who do not, although our results did not provide clear evidence for an actual difference in age-related AMH trajectories. Future studies that investigate the association between age-specific AMH (trajectories) and type 2 diabetes should ideally include a larger proportion of younger women and, if possible, include proxies for biological ageing.

## Electronic supplementary material

ESM (PDF 401 kb)

## Data Availability

The full dataset and statistical codes are available on request, in liaison with the National Institute of Public Health and the Environment.

## Abbreviations

AMH: Anti-Müllerian hormone
CG–LMS: Cole and Green, Lambda, Mu and Sigma
PCOS: Polycystic ovary syndrome
HRT: Hormone replacement therapy
