# Supplementary material for: Anti-Müllerian hormone levels and risk of type 2 diabetes in women
Source: Diabetologia. 2020 Oct 13;64(2):375–84. doi: 10.1007/s00125-020-05302-5 (PMC7801305; doi:10.1007/s00125-020-05302-5)
Supplement: Supplementary file 1 — (PDF 401 kb) [file 125_2020_5302_MOESM1_ESM.pdf]

**Electronic supplementary material (ESM) corresponding to *Anti-Müllerian Hormone Levels and Risk of Type 2 Diabetes in Women***

## ESM Methods

Missing values in baseline age-specific AMH tertiles and baseline and time-varying covariates and were imputed with multiple imputation (100 iterations, 10 imputed datasets) using the R package “mice”. Imputation models were dependent on the type of variable: predictive mean matching, logistic regression, and ordered logit models were used for continuous (BMI, alcohol consumption and total cholesterol), binary (current smoking, physical activity, hypertension, current oral contraceptive use, menopausal status, ever hormone replacement therapy use), and ordinal categorical (age-specific AMH tertiles, educational attainment) variables, respectively. Predictor variables were selected based on their presence in subsequent analyses, their mutual correlations and their correlation with the imputed variables. For imputation of repeated variables we used variables for the previous and following examination rounds as predictors.

**ESM Table 1.** Baseline characteristics presented by availability of AMH measurements per round.

| Baseline characteristics | Round 1 | | Round 2 | | Round 3 | | Round 4 | | Round 5 | |
|---|---|---|---|---|---|---|---|---|---|---|
| | Women with AMH measurement at Round 1 (*n* = 3104) | Women without AMH measurement at Round 1 (*n* = 189) | Women with AMH measurement at Round 2 (*n* = 2888) | Women without AMH measurement at Round 2 (*n* = 405) | Women with AMH measurement at Round 3 (*n* = 2488) | Women without AMH measurement at Round 3 (*n* = 805) | Women with AMH measurement at Round 4 (*n* = 2305) | Women without AMH measurement at Round 4 (*n* = 988) | Women with AMH measurement at Round 5 (*n* = 2038) | Women without AMH measurement at Round 5 (*n* = 1255) |
| Age, years[a] | 39.5 (32.0, 47.9) | 37.9 (31.3, 44.7) | 39.5 (32.2, 47.4) | 38.2 (30.2, 48.5) | 39.0 (31.8, 46.8) | 41.0 (32.9, 51.0) | 38.5 (31.6, 46.3) | 41.5 (33.1, 51.3) | 37.7 (31.3, 45.6) | 41.8 (33.8, 51.4) |
| BMI, kg/m$^2$[a] | 23.6 (21.6, 26.1) | 23.8 (21.6, 26.5) | 23.6 (21.6, 26.1) | 23.7 (21.6, 26.1) | 23.5 (21.6, 25.9) | 24.1 (21.8, 26.9) | 23.3 (21.4, 25.7) | 24.3 (21.9, 27.4) | 23.2 (21.4, 25.5) | 24.3 (22.0, 27.0) |
| Educational attainment, % | | | | | | | | | | |
| Low | 68.0 | 71.8 | 68.3 | 67.9 | 65.5 | 76.8 | 63.9 | 78.3 | 61.5 | 79.2 |
| Middle | 19.0 | 19.1 | 18.9 | 20.0 | 20.1 | 15.6 | 21.1 | 14.3 | 22.7 | 13.1 |
| High | 13.0 | 9.0 | 12.8 | 12.1 | 14.4 | 7.6 | 15.0 | 7.4 | 15.8 | 7.7 |
| *Reproductive factors* | | | | | | | | | | |
| Parous, % | 76.8 | 68.8 | 76.7 | 73.6 | 76.4 | 76.1 | 75.7 | 77.9 | 75.6 | 77.6 |
| Postmenopausal, % | 15.1 | 15.0 | 15.0 | 16.0 | 13.8 | 19.4 | 12.6 | 21.2 | 10.8 | 22.5 |

| | | | | | | | | | | |
|---|---|---|---|---|---|---|---|---|---|---|
| Current OC use, % | 25.4 | 30.2 | 25.0 | 30.3 | 26.3 | 23.5 | 26.6 | 23.5 | 27.6 | 22.5 |
| *Lifestyle factors* | | | | | | | | | | |
| Current smoker, % | 33.7 | 37.6 | 34.0 | 32.9 | 31.8 | 40.5 | 31.3 | 40.0 | 29.9 | 40.3 |
| Current alcohol consumption, % | | | | | | | | | | |
| No | 19.4 | 19.1 | 19.3 | 20.3 | 17.4 | 25.3 | 17.3 | 24.2 | 16.8 | 23.5 |
| <1 glass/week | 31.0 | 33.5 | 30.9 | 32.7 | 31.5 | 30.0 | 31.8 | 29.6 | 32.0 | 29.8 |
| ≥1 glass/week | 49.6 | 47.3 | 49.8 | 47 | 51.1 | 44.6 | 50.9 | 46.1 | 51.3 | 46.6 |
| Physically active, % | 72.0 | 65.3 | 71.9 | 61.3 | 73.6 | 66.1 | 73.6 | 67.2 | 74.3 | 67.4 |
| Total cholesterol, mmol/l[a] | 5.3 (4.6, 6.0) | 5.3 (4.6, 6.1) | 5.3 (4.6, 6.0) | 5.2 (4.6, 6.0) | 5.2 (4.6, 6.0) | 5.3 (4.7, 6.1) | 5.2 (4.6, 5.9) | 5.5 (4.8, 6.2) | 5.2 (4.5, 5.8) | 5.5 (4.8, 6.3) |
| Hypertension, % | 13.5 | 15.9 | 13.6 | 13.8 | 12.4 | 17.6 | 11.0 | 19.8 | 9.6 | 20.2 |
| *Outcome* | | | | | | | | | | |
| Incident diabetes, % | 4.8 | 7.9 | 4.8 | 5.9 | 5.5 | 3.4 | 5.5 | 3.6 | 5.3 | 4.4 |

[a] Median (IQR)

AMH, anti-Müllerian hormone; BMI, body mass index; OC, oral contraceptive

**ESM Table 2.** Fixed effects of linear mixed models with change in $_{log}$AMH (pmol/l) over age as outcome.

| | | Total study population (*n* = 3290, 162 cases) | Exclusion of AMH measurements within 2 years prior to type 2 diabetes diagnosis (*n* = 3279, 151 cases) | Exclusion of women who reported ever to have used HRT[a] (*n* = 1801, 95 cases) | Exclusion of women who potentially had PCOS (*n* = 3023, 138 cases) |
|---|---|---|---|---|---|
| | | | | | |
| | | **Estimate (95% CI)** | **Estimate (95% CI)** | **Estimate (95% CI)** | **Estimate (95% CI)** |
| **Fixed intercept** | | 5.64 (5.16, 6.12) | 5.66 (5.17, 6.15) | 6.05 (5.43, 6.66) | 5.74 (5.24, 6.24) |
| **T2D vs. no T2D** | | -1.29 (-3.32, 0.74) | -1.09 (-3.14, 0.96) | -0.71 (-2.73, 1.30) | -1.55 (-3.63, 0.53) |
| **Age** | | | | | |
| | **1st spline** | -5.74 (-5.91, -5.56) | -5.73 (-5.91, -5.56) | -5.53 (-5.72, -5.34) | -5.71 (-5.89, -5.54) |
| | **2nd spline** | -3.05 (-3.46, -2.65) | -3.06 (-3.46, -2.65) | -3.25 (-3.72, -2.79) | -3.17 (-3.58, -2.76) |
| | **3rd spline** | -6.31 (-6.48, -6.14) | -6.31 (-6.47, -6.14) | -6.09 (-6.29, -5.90) | -6.32 (-6.49, -6.15) |
| **1st spline * T2D** | | 0.55 (-0.69, 1.80) | 0.53 (-0.75, 1.80) | 0.38 (-0.86, 1.62) | 0.83 (-0.44, 2.10) |
| **2nd spline * T2D** | | 2.42 (-1.65, 6.48) | 2.01 (-2.11, 6.12) | 1.14 (-2.99, 5.27) | 2.97 (-1.21, 7.15) |
| **3rd spline * T2D** | | 0.51 (-0.45, 1.47) | 0.33 (-0.66, 1.33) | 0.31 (-0.64, 1.26) | 0.60 (-0.39, 1.58) |
| **P-value likelihood ratio tests** | | 0.58 | 0.67 | 0.84 | 0.65 |

Models additionally included the following fixed effects: current oral contraceptive use, current smoking, body mass index, menopausal status, alcohol consumption, physical activity, hypertension, total cholesterol, parity and educational level.

[a] Numbers differed between imputation sets, as the variable ever HRT use itself was imputed; presented numbers are averages.
AMH, anti-Müllerian hormone; HRT, hormone replacement therapy; PCOS, polycystic ovary syndrome
